# Biofilm Producing Methicillin-Resistant *Staphylococcus aureus* (MRSA) Infections in Humans: Clinical Implications and Management

**DOI:** 10.3390/pathogens13010076

**Published:** 2024-01-15

**Authors:** Ashlesha Kaushik, Helen Kest, Mangla Sood, Bryan W. Steussy, Corey Thieman, Sandeep Gupta

**Affiliations:** 1 Division of Pediatric Infectious Diseases, St. Luke’s Regional Medical Center, Unity Point Health, 2720 Stone Park Blvd, Sioux City, IA 51104, USA; 2 Department of Pediatrics, University of Iowa Carver College of Medicine, Iowa City, IA 52242, USA; 3 Master of Science, Healthcare Quality and Safety, Harvard Medical School, Boston, MA 02115, USA; 4 Division of Pediatric Infectious Diseases, St. Joseph’s Children’s Hospital, 703 Main Street, Paterson, NJ 07503, USA; kesth@sjhmc.org; 5 Department of Pediatrics, Indira Gandhi Medical College, Shimla 171006, India; drmanglasood@gmail.com; 6 Division of Microbiology, St. Luke’s Regional Medical Center, Unity Point Health, 2720 Stone Park Blvd, Sioux City, IA 51104, USA; bryan.steussy@unitypoint.org; 7 Division of Pharmacology, St. Luke’s Regional Medical Center, Unity Point Health, 2720 Stone Park Blvd, Sioux City, IA 51104, USA; corey.thieman@unitypoint.org; 8 Division of Pulmonary and Critical Care, St. Luke’s Regional Medical Center, Unity Point Health, 2720 Stone Park Blvd, Sioux City, IA 51104, USA; sandeep.gupta@unitypoint.org

**Keywords:** methicillin-resistant *Staphylococcus aureus*, MRSA, biofilm, infection, treatment, antibiotics

## Abstract

Since its initial description in the 1960s, methicillin-resistant *Staphylococcus aureus* (MRSA) has developed multiple mechanisms for antimicrobial resistance and evading the immune system, including biofilm production. MRSA is now a widespread pathogen, causing a spectrum of infections ranging from superficial skin issues to severe conditions like osteoarticular infections and endocarditis, leading to high morbidity and mortality. Biofilm production is a key aspect of MRSA’s ability to invade, spread, and resist antimicrobial treatments. Environmental factors, such as suboptimal antibiotics, pH, temperature, and tissue oxygen levels, enhance biofilm formation. Biofilms are intricate bacterial structures with dense organisms embedded in polysaccharides, promoting their resilience. The process involves stages of attachment, expansion, maturation, and eventually disassembly or dispersion. MRSA’s biofilm formation has a complex molecular foundation, involving genes like *icaADBC*, *fnbA*, *fnbB*, *clfA*, *clfB*, *atl*, *agr*, *sarA*, *sarZ*, *sigB*, *sarX*, *psm*, *icaR*, and *srtA*. Recognizing pivotal genes for biofilm formation has led to potential therapeutic strategies targeting elemental and enzymatic properties to combat MRSA biofilms. This review provides a practical approach for healthcare practitioners, addressing biofilm pathogenesis, disease spectrum, and management guidelines, including advances in treatment. Effective management involves appropriate antimicrobial therapy, surgical interventions, foreign body removal, and robust infection control practices to curtail spread within healthcare environments.

## 1. Introduction

Methicillin-resistant *Staphylococcus aureus* (MRSA) has emerged as a highly formidable pathogen in contemporary times, causing significant levels of illness and death due to its ability to counteract immune defenses through various mechanisms. First identified in the 1960s, MRSA has evolved to develop numerous mechanisms of antimicrobial resistance and evasion of the host’s immune system [1,2]. This enables MRSA to cause invasive diseases, including those involving biofilm formation. With its diverse arsenal of evasion strategies against the host’s defenses, MRSA has become a pervasive pathogen responsible for a range of infections. These infections span from chronic and recurring skin and soft tissue infections (SSTIs) to more deeply-seated conditions such as infections of the bones and joints (osteoarticular infections) and endocarditis, leading to substantial morbidity and mortality [3,4,5,6].

Biofilm production contributes to the persistence of infections, as strains exhibiting enhanced virulence thrive within these protective structures [7]. In the pursuit of improved treatments, numerous molecules are being explored as supplementary therapies to antibiotics, representing a vibrant area of ongoing research. This review aims to describe the pathogenesis, clinical implications, and current state-of-the-art treatment for MRSA biofilm infections in humans as well as address the possible role of newer and adjunctive therapies for combating biofilms.

## 2. MRSA Biofilm: Pathogenesis

First acknowledged in the 17th century by Antoni van Leeuwenhoek, a dry goods merchant, biofilms were described as “microorganisms” that exhibit swarming tendencies in both inanimate and living substrates, resisting aggressive and meticulous cleaning procedures [8]. The process of biofilm formation is an innate and indispensable element within the prokaryotic life cycle. Its presence augments survival probabilities in inhospitable habitats, thereby facilitating persistence, continuity, and dispersion to foster the establishment of novel ecological niches [7,9]. Although first recorded by Henrici in 1933, a recent publication by the National Institute of Health suggested that almost 60% of all infections in in vivo conditions are caused by bacteria embedded in biofilms [10,11].

Biofilm formation requires a suitable substrate or surface and environment. For example, colonization and subsequent biofilm production occurs more frequently on rough surfaces because of the higher surface area and favorable physicochemical properties [12,13,14]. This means that implanted medical device materials or biomaterials can be easy targets with different potential for biofilm development. Similarly, the rate and extent of adherence vary depending on the composition of chemicals that coat the biofilm [15,16,17]. Staphylococci, including MRSA, are known to be the leading cause of infections linked to biofilms [18].

*S. aureus* biofilms mainly consist of water and organic elements. Bacterial microcolonies and extracellular polymeric substance (EPS) form a considerable part of biofilm [19]. EPS is a blend of diverse polymeric materials, including polysaccharides, extracellular DNA (eDNA), and proteins.

Biofilm formation takes place in multiple stages, including attachment, formation/maturation, and dispersal [20]. The initial attachment of a bacterium to biotic (endovascular, bone, or joint) or abiotic (prosthetic device/catheter) surfaces involves a number of proteins known as Microbial Surface Components Recognizing Adhesive Matrix Molecules (MSCRAMMs) [21]. The main Microbial Surface Component Recognizing Adhesive Matrix Molecule proteins involved in *S. aureus* adhesion are clumping factor A (ClfA) and clumping factor B (ClfB) [22]. These clumping factor proteins guide initial attachment by binding to fibrinogen [18]. Other adhesive Microbial Surface Components Recognizing Adhesive Matrix Molecules in the *S. aureus* arsenal include bone sialo-binding protein (Bbp) which attaches to the extracellular matrix and collagen adhesin (Can) which binds collagen [22]. Alpha enolase is another such protein involved in initial attachment that is encoded by the *eno* gene. It is involved in the attachment of *S. aureus* to the extracellular matrix by binding plasminogen [23]. This ability to bind plasminogen and fibrinogen promotes MRSA colonization in sites of injury [23]. Following attachment and initial growth of the microcolonies the biofilm begins to mature by secretion of the extracellular polymeric substance (EPS). 

In *S. aureus* biofilm, the major component of EPS is the polysaccharide intercellular adhesin (PIA). PIA is a cationic polysaccharide composed of partially deacetylated N-acetylglucosamine monomers. Its biosynthesis is orchestrated by the genetic elements within the *ica* (regulatory locus) and *icaADBC* (the synthesizing locus) [24,25]. MRSA can also form biofilms independent of *icaADBC* mechanisms [26]. Biofilm accumulation can occur through the action of the *icaADBC*-encoded polysaccharide intercellular adhesin or by *S. aureus* surface proteins such as Bap, SasG, SasC, fibronectin-binding proteins, and protein A [26,27]. PIA fulfills a substantial role in the formation of staphylococcal biofilms and the evasion of host immune responses, including imperviousness to phagocytic ingestion and eradication and the propensity to enhance infectivity. The presence of the *ica* genes correlates with heightened pathogenic potential [18]. The EPS, mainly due to the contribution of PIA, is made up predominantly of polysaccharides [28], although it is also composed of other proteins, lipids, and extracellular DNA [29]. The EPS allows for antimicrobial resistance both physically by separating the individual bacteria from the antimicrobial compounds, but also by allowing the bacteria to decrease their metabolic activity which can also contribute to the antimicrobial resistance [30].

The biofilm matures as the *S. aureus* interacts with the EPS utilizing proteins such as Aap [17]. Subsequent to its formation, the biofilm can undergo dispersal mediated by mechanical influences and the excretion of enzymatic and chemical agents. These agents facilitate the breakdown of the extracellular polymeric matrix, enabling segments of the biofilm to disperse and establish new colonization sites [19]. The main genes encoding the dispersal are regulated by the accessory gene regulatory *(agr)* operon system that encodes AgrA, AgrB, AgrC, and AgrD and induces dispersal via proteases and phenol-soluble modulins [19]. The “decision” of a biofilm to initiate the secretion of substances causing dispersion can stem from a range of influences, encompassing the accessibility of nutrients and oxygen, the existence of toxins, and additional stressors [31]. This decision-making process is governed by the accessory gene regulator (*agr*) system, which concurrently regulates quorum sensing, a mechanism of cellular communication. This *agr* system comprises paired genes that become active upon recognition of autoinducing peptides [32]. The autoinducing peptides, which are a product of the AgrD encoding process, amass and subsequently trigger the activation of the paired genes. This autoinducing peptide holds promise as a potential target for therapeutic interventions aimed at disrupting biofilm formations [32]. Dispersion of a biofilm can occur via two forms: active or passive dispersion. 

During active dispersion, bacteria within the biofilm release enzymes, particularly phosphodiesterases, which are essential for breaking down the extracellular matrix to facilitate dispersion of bacterial microcolonies [33]. This phenomenon is prompted by alterations in the surrounding environment. Passive dispersion transpires through either mechanical means or the external enzymatic breakdown of biofilm structures [10,33]. Once dispersed, the planktonic bacteria are free to re-colonize and begin the process over again [19]. 

## 3. Clinical Implications

Biofilm-associated MRSA can cause a broad range of infections ranging from SSTIs to deep-seated infections including blood stream infections (BSIs), osteomyelitis, and infective endocarditis (IE). MRSA infections are a significant concern in intensive care units, where many strains have developed resistance to various antibiotics. These infections are particularly troubling due to their association with hospital settings and the substantial morbidity and mortality they cause [4,6,34]. Adding to the complexity, the adoption of a biofilm state by MRSA where it is encased within a self-produced extracellular matrix allows it to adhere to a wide range of surfaces. Biofilm formation is a crucial factor in the virulence of *Staphylococcus* bacteria, leading to prolonged and recurring infections linked to medical devices. MRSA biofilms can develop on various devices like catheters, contact lenses, mechanical heart valves, and prostheses.

Microorganisms in biofilms have the ability to enter a dormant state for extended periods, ranging from weeks to years. This latent condition can persist until conducive conditions arise, leading to the manifestation of localized or systemic indications and symptoms of infection. Recurring infections after repeated antibiotic interventions is commonly attributed to the presence of biofilms [7,11]. Because of the structure and polymeric matrix, biofilms provide a unique protective mechanism that enable bacteria to evade antibiotics by preventing antibiotic diffusion, thus leading to the emergence of multidrug-resistant populations of bacteria. Bacteria residing within biofilms exhibit antibiotic resistance levels up to 1000 times greater than their planktonic counterparts [35]. Multidrug-resistant infections pose a great challenge for antibiotic therapy and eradication of infections: the main risk factor associated with MRSA as the cause of some health care associated infections is prior 90 days’ intravenous antibiotics use. In addition to antibiotic resistance, by offering protection against environmental stressors (such as sheer forces, drying) and phagocytosis, biofilms play a critical role in various infections including skin and soft tissue infections, medical-device-related infections, and catheter-associated intravascular or urinary tract infections [36,37].

MRSA colonization is a significant step in the development of active MRSA infections and a key factor in the spread of MRSA within healthcare settings. This colonization substantially heightens the risk of MRSA infection acquisition during hospitalization. Biofilms have been implicated as a major factor in both MRSA colonization and the challenges encountered in successfully eradicating MRSA colonization [38,39,40].

A substantial proportion of individuals carrying community-associated MRSA exhibit colonization at bodily sites beyond the anterior nasal passages. For instance, a study found that 23% of patients with community-associated MRSA colonization showed colonization in non-nasal regions, predominantly in the inguinal areas. Additionally, in children with skin and skin structure infections (SSSI), the rectum emerged as the primary colonization site [41]. Even within the intensive care unit context, MRSA carriage is notable in the throat and rectum. Recent evidence suggests that community-associated MRSA carriage patterns may deviate from the established norms of healthcare-associated MRSA. Burn injuries, in particular, experience rapid colonization by Gram-positive bacteria, mainly *S. aureus*, originating from the patient’s skin and contaminated surfaces. 

A significant majority (approximately 90%) of *S. aureus* infections fall under the category of skin and skin structure infections (SSSI), making up the primary portion of staphylococcal disease [3]. However, infections affecting the bloodstream, respiratory tract, bones, joints, surgical wounds, and increasingly medical devices provoke heightened concern due to their higher rates of illness and death, necessitating prolonged treatment periods [4,5,6,34,42,43]. More recently, severe forms of community-associated infections, including fulminant sepsis, the Waterhouse–Friderichsen syndrome, and necrotizing pneumonia, have emerged as prominent issues. The escalation of antibiotic resistance has further compromised the effectiveness of existing antimicrobial agents [44,45].

One of the most important manifestations of *S. aureus* infection is bacteremia. MRSA bacteremia can be divided into two categories: uncomplicated and complicated. In the uncomplicated category, cases exhibit positive blood cultures, the exclusion of infective endocarditis (IE), the absence of implanted prostheses, negative follow-up blood cultures 48 h after initial positive blood cultures, defervescence within 72 h of appropriate antibiotic therapy, and the absence of metastatic sites of infection. In contrast, complicated MRSA bacteremia cases show positive blood cultures in conjunction with any of the aforementioned criteria [46,47]. Roughly 43% of *S. aureus* bacteremia cases fall into this complicated category. Common primary sources leading to bacteremia include vascular catheter-related infections, skin and soft tissue infections, pleuropulmonary infections, osteoarticular infections, and infective endocarditis (IE). Notably, up to 25% of MRSA bacteremia cases lack an identifiable focus of infection. Primary *S. aureus* bacteremia has high rates of metastatic foci of infection, most commonly infective endocarditis (IE). Therefore, transesophageal or transthoracic echocardiogram is recommended for all patients with MRSA bacteremia [47,48]. Those at particularly high risk for secondary IE include patients with prosthetic valves, implanted cardiac device such as pacemakers, previous history of IE, and intravenous drug users. 

*S. aureus* is the most common cause of osteomyelitis across all age groups [49]. In children, the primary mechanism of osteomyelitis involves hematogenous spread [42,50]. Infection is usually monomicrobial and primarily affects long bones. In contrast, in adults, contiguous osteomyelitis (associated with other primary infections adjacent to the affected bone such as cellulitis, septic arthritis, or overlying chronic wound) is more common than hematogenous osteomyelitis. Hematogenous osteomyelitis in adults is most commonly seen in the thoracic and lumbar vertebrae [51]. Hematogenous MRSA prosthetic joint infection (HMPJI) is more commonly associated with community-acquired infection and rarely with nosocomial infection. Risk factors associated with the acquisition of HMPJI include the presence of three or more arthroplasties, where knee arthroplasty is a greater risk than hip arthroplasty, and a history of arthroplasty revision [52].

## 4. Management of Biofilm Infections

Suggested approaches for management of *S. aureus* biofilm infections include treatment by antimicrobials alone/in combination along with surgical debridement or device removal, inhibiting biofilm formation by limiting bacterial attachment to medical devices with surface modification, application of newer anti-biofilm agents, or novel technologies like laser Shock waves (LSW). 

While several novel strategies for treatment/inhibition of biofilms are being investigated, the most commonly used approaches for management of biofilm producing MRSA infections currently center around prolonged use of appropriate antibiotics and removal of sources/foci of infection/foreign bodies like catheters [46,53]. The various management strategies are discussed below, including the use of antimicrobials as the first-line therapy for biofilm infections, and the following section discusses the current treatment recommendations for the disease spectrum of MRSA infections in adults and pediatric populations. 

### 4.1. Current Guidelines for Treatment of MRSA Infections

Antibiotics represent the cornerstone of therapy for infections caused by MRSA strains known for their biofilm-forming capabilities. The Infectious Disease Society of America (IDSA) guidelines address management of MRSA infections including skin and soft tissue infections, bacteremia, infective endocarditis, pneumonia, osteomyelitis, joint infections, and central nervous system infections, all of which are facilitated by biofilm production [46]. When managing MRSA infections, the IDSA strongly recommends debriding and draining any soft tissue abscesses associated with the infection whenever possible, in addition to initiation of antimicrobials.

Detailed information regarding antimicrobial agents with demonstrated efficacy against MRSA can be found in Table 1 and Table 2 [46,54,55,56]. The anti-MRSA antimicrobials include vancomycin, daptomycin, linezolid, trimethoprim–sulfamethoxazole, clindamycin, rifampin, long-acting agents like dalbavancin, oritavancin, and telavancin, and tetracyclines such as doxycycline, minocycline, and tigecycline, with some other novel antimicrobials like omadacycline (a tetracycline), lefamulin, and delafloxacin (the first fluoroquinolone with anti-MRSA activity) only approved for certain disease states like SSTIs or pneumonia [46,54]. Table 1 summarizes the different antimicrobials for MRSA infections and provides an overview of the pharmacokinetic properties, mechanism of action, and adverse drug reaction profiles of these agents. Dosing of antimicrobials for MRSA infections in children and adults are presented in Table 2. While antimicrobial monotherapy involving anti-MRSA agents may suffice for uncomplicated infections, the management of more complex scenarios, such as prosthetic valve endocarditis, may necessitate a dual-therapy approach. Furthermore, comprehensive management strategies for all MRSA infections should encompass the rigorous identification, elimination, and, where feasible, surgical debridement of the primary infectious foci, as well as any additional sites of infection.

Recommended antibiotics for the treatment of MRSA osteomyelitis include vancomycin, daptomycin, or linezolid, with some experts recommending additional rifampin therapy. Duration of therapy is also important and an individual with osteomyelitis should receive at least 8 weeks at minimum and possibly 3 months or longer of therapy [46,54]. After an initial intravenous therapy course, patients with MRSA osteomyelitis should be transitioned to oral therapy and some experts suggest rifampin with any of the following based on susceptibilities: trimethoprim–sulfamethoxazole (TMP-SMX), a tetracycline derivative, or clindamycin [46]. 

Similar to the treatment of osteomyelitis without device involvement, managing patients with osteoarticular infections related to medical devices follows similar antimicrobial therapy guidelines, with the inclusion of combination therapy involving rifampin [46]. Patients who develop an infection within 2 months after surgery or those with a stable implant and hematogenous infection should receive the aforementioned parenteral therapy in combination with rifampin for a duration of 2 weeks.

For patients with hip involvement, an additional 3 months of oral clindamycin, TMP-SMX, a fluoroquinolone, or a tetracycline in combination with rifampin is warranted. Patients with prosthetic knee infections should receive an extended course of similar oral therapy lasting an additional 6 months. Debridement is also strongly recommended with device retention in stable situations and consideration for device removal in unstable situations [46]. 

The treatment of osteomyelitis in pediatric populations diverges from adult recommendations [55]. In children aged 4 months to 18 years, vancomycin monotherapy is the indicated treatment. However, for isolates that are sensitive, second-line options such as linezolid, daptomycin, TMP-SMX, or clindamycin may be considered [55]. Newborns under 4 months of age should be treated with either vancomycin or linezolid. The recommended treatment duration for pediatric osteomyelitis is four to six weeks. 

*S. aureus* is one of the more common bacteria associated with vertebral osteomyelitis and empiric therapy for this condition should include MRSA coverage. IDSA MRSA treatment guidelines recommend treatment regimens that include vancomycin and either ceftriaxone, cefepime, or levofloxacin for additional Gram-negative coverage [46]. Alternative recommended MRSA antimicrobial agents include daptomycin or linezolid. Treatment of vertebral osteomyelitis is usually prolonged with patients needing antibiotics for a total duration of 8 weeks or more [46]. For patients with spinal implant infections occurring less than or equal to 30 days after an implant procedure, a similar initial dosing strategy is recommended. Parenteral therapy including rifampin is recommended with a transition to oral coverage including dual oral therapy with rifampin. It is recommended to continue oral therapy until spinal fusion has occurred. For patients experiencing an infection greater than 30 days after implant procedure, device removal is recommended with a similar antimicrobial treatment strategy.

MRSA endocarditis treatment recommendations depend on the presence of a native or mechanical cardiac valve. In patients with infective endocarditis without a prosthetic valve, vancomycin or daptomycin, both as monotherapy, are recommended for an extended course of 4–6 weeks [46]. In pediatric patients, vancomycin is the drug of choice for infective endocarditis, and daptomycin may be considered as an alternative [46]. When a prosthetic cardiac valve is present, a combination therapy regimen approach is recommended, with vancomycin and rifampin administered for a total of 6 weeks with the addition of low dose gentamicin for the first 2 weeks of treatment.

IDSA guidelines recommend managing MRSA meningitis with intravenous vancomycin for a total of 2 weeks [46]. Some experts recommend adding rifampin to this regimen. Other treatment options include linezolid or TMP-SMX. If a CNS shunt is present, removal of the device is strongly recommended. Guidelines recommend leaving the shunt out until cerebrospinal fluid cultures are repeatedly negative. Pediatric patients diagnosed with MRSA meningitis should receive vancomycin alone.

Due to the complex nature of these infections and the principles of pharmacokinetics, such as drug distribution and concentration levels in various tissues, dosing strategies for vancomycin and daptomycin in these patients are more aggressive. Vancomycin has traditionally been dosed based on actual body weight, with a range of 15–20 mg/kg per dose administered every 8–12 h, not exceeding 2 g per dose [46]. Although traditionally vancomycin trough concentrations have been used for vancomycin monitoring with target trough concentrations for serious infections between 15–20 μg/mL, the American Society of Health-System Pharmacists, the Infectious Diseases Society of America, the Pediatric Infectious Diseases Society, and the Society of Infectious Diseases Pharmacists revised their vancomycin dosing guidelines in 2020, recommending using the Bayesian-derived AUC (area under the curve)/MIC (minimal inhibitory concentration) ratio for vancomycin monitoring instead of trough concentrations in order to achieve optimal drug efficacy and reduce the risk of acute kidney injury [56]. In general, according to these guidelines, vancomycin target AUC goals of 400 and 600 mg × h/L are desired in patients with a confirmed MRSA diagnosis and MRSA isolates with MIC value of ≤1 mg/L [40]. For critically ill adult patients, the guidelines recommend a vancomycin dosing approach that includes a 20–35 mg/kg loading dose with a maximum not to exceed 3 g before initiating a pharmacokinetic-based calculated regimen. The guidelines recommend monitoring of AUC levels early in the course of treatment (24–48 h) [56].

In patients with MIC values greater than 1 mg/L, alternatives to vancomycin therapy should be considered, as treating MRSA isolates with an MIC > 1 mg/L requires higher doses of vancomycin to achieve desired AUC goals and increases the risk of toxicities. Daptomycin doses are also generally higher for these indications (8–10 mg/kg and occasionally 12 mg/kg every 24 h).

MRSA bacteremia remains an ongoing treatment challenge for practitioners with treatment failure associated with poor patient outcomes. Further investigation of the impact of both monotherapy and dual-therapy treatment regimens on clinical success rates is warranted [57]. For MRSA bacteremia, combination therapies have been utilized, especially in case of resistance to daptomycin and vancomycin. A literature review by Lewis et al. evaluated case-study reviews of antimicrobial regimens in patients with MRSA bacteremia [57]. Findings included daptomycin in combination with anti-*Staphylococcal* beta-lactam antibiotics such as nafcillin, oxacillin, and ceftaroline showing improved clinical success rates in persistent MRSA bacteremia. Based on recent studies, it has been recommended that if repeat blood cultures fail to become negative at 3–5-days despite appropriate antibiotic therapy, the patient should be considered to have monotherapy failure, prompting the addition of ceftaroline to vancomycin or switching to daptomycin with a second antimicrobial agent [58,59]. Daptomycin has been successfully used in combination with rifampin or trimethoprim–sulfamethoxazole to treat MRSA bacteremia [60,61]. Similarly, combination therapy including vancomycin-based regimens with anti-*Staphylococcal* beta-lactams has shown to be potentially useful [62,63].

According to IDSA guidelines, vancomycin, gentamicin, and rifampin remain the standard of care for staphylococcal prosthetic valve endocarditis [46]. Rifampin in particular shows a strong ability to permeate biofilms and hence bactericidal activity against biofilm-producing microbes that are susceptible. In a study, rifampin in combination with daptomycin was demonstrated to be a successful regimen in treating persistent MRSA infections commonly involving biofilm formation in 10 of 12 patients [60]. In fact, IDSA guidelines recommend using rifampin in conjunction with other antibiotics for MRSA infections in prosthetic joints, infective endocarditis on prosthetic valves, and ventriculitis and meningitis with hardware [46,64,65,66,67,68]. Dosing for rifampin for biofilm-associated *S aureus* infections in a pediatric population range from 10 mg/kg/d to 20 mg/kg/d, given in 1 to 3 doses, with a maximum of 600 mg per dose and 900 mg/d [46]. Other combinations that have shown promise include ceftaroline alone or combined with trimethoprim–sulfamethoxazole or vancomycin [69,70], combinations of linezolid with a carbapenem, or telavancin with ceftaroline or rifampin [71,72]. Quinupristin–dalfopristin can also be used as a salvage therapy agent; however, it is not preferred given the adverse effect profile.

Thus, to summarize, dual antimicrobial therapy must be considered, especially while treating critical MRSA infections with hardware such as endocarditis, central nervous system infections, or osteomyelitis. Most of these combinations include rifampin with its property of biofilm penetration.

For MRSA infection prevention and control, isolation and cohorting of MRSA infected or colonized patients during hospitalization are recommended. Guidelines emphasize following contact precautions and meticulous hand hygiene during care of these patients for preventing spread and transmission of MRSA infections. In addition, MRSA decolonization measures including mupirocin application to nares, topical cleansing with chlorhexidine, and cleaning of frequently touched surfaces, are recommended in order to prevent recurrent MRSA infections [46].

Various methods are employed to determine if *S. aureus* isolates are susceptible to different antimicrobials, including phenotypic and genotypic techniques [73,74]. According to the Centers for Disease Control and Prevention, MRSA are *S. aureus* strains that are oxacillin- and methicillin-resistant, and are resistant to all ß-lactam antibiotics with the exception of 5th generation MRSA-active cephalosporins like ceftaroline [73]. Phenotypic approaches include disk diffusion, micro- and macro-dilution, and epsilometer tests, where the microbes are exposed to various concentrations of an antibiotic and the effects on the bacteria are directly observed. Methods used for testing for MRSA include the Clinical and Laboratory Standards Institute (CLSI) recommended methods, like the broth microdilution testing, cefoxitin disk diffusion test, or a plate containing 6 μg/mL of oxacillin in Mueller–Hinton agar supplemented with 4% NaCl [73,74]. Resistance is detected through minimum inhibitory concentration (MIC) or zone diameter break point (ZDP) values [75]. For instance, when testing methicillin on a *S. aureus* isolate, ZDP of ≤9 mm or an MIC ≥ 16 µg/mL would indicate the isolate is resistant to methicillin and should be designated MRSA, as classified by the CLSI [74]. Alternatively, a ZDP of ≥14 mm or an MIC of ≤8 µg/mL indicates a methicillin-susceptible *S. aureus* isolate and therapy should be de-escalated to MSSA-appropriate therapy such as nafcillin, oxacillin, methicillin, or cefazolin [54,74]. *S. aureus* isolates resistant to methicillin are considered resistant to oxacillin and vice versa. Strains considered susceptible to either medication are considered susceptible to the other. Genotypic detection methods include PCR and DNA microarrays where the resistance is identified through gene detection [75]. MRSA specifically expresses the *mecA* gene which produces a low-affinity penicillin-binding protein that confers β-lactam antibiotic resistance to the microbe. Simultaneous detection of *S. aureus* and the *mecA* gene classify the bacteria as MRSA, whereas detection of *S. aureus* without *mecA* would be designated as an MSSA isolate potentially warranting a de-escalation of antimicrobial therapy [54]. Since 1996, MRSA strains with decreased susceptibility to vancomycin have been described. These include strains labeled as vancomycin intermediate *S. aureus* (VISA), defined as those with MIC between 4–8 μg/mL, and strains fully resistant to vancomycin labeled as vancomycin-resistant *S. aureus* (VRSA) with vancomycin MIC ≥ 16 μg/mL [73]. These can be challenging to treat, often requiring antimicrobials like daptomycin, linezolid, and telavancin [54].

### 4.2. Ethanol Locks, Antibiotic Lock Therapy, and Coated Implants to Inhibit Biofilms

In addition to antibiotics with infection-source control as the first line therapy, other strategies to prevent biofilm mediated device infections have been suggested. Alcohol lock therapy may be of some benefit as shown by some studies [76,77]. Ethanol has been demonstrated to have high anti-biofilm activity, is easy to use, and is inexpensive, without reports of resistance with successful catheter salvage rates of >70% [76,77]. In one study, heparinized 40% ethanol lock solution significantly reduced bacterial metabolic activity; however, it was not able to eradicate the biofilm completely [76]. Despite these advantages, some disadvantages include lack of consensus guidance around exact dosing, timing/combinations with anticoagulants, several notable adverse effects including, catheter occlusion, plasma protein precipitation, and risk of thrombosis [77,78], and concerns about abnormalities in catheter integrity, including one case leading to catheter embolization [77].

Antibiotic lock therapy with daptomycin, minocycline alone, and minocycline in combination with rifampin have been studied; however, these therapies are not preferred given the concern for rapid emergence of antimicrobial resistance. In a study, authors showed that antibiotics, especially daptomycin and minocycline, were effective in MRSA eradication when employed as lock therapy [79]. The study showed that after 3 days of 4-h every-day exposure, daptomycin, minocycline, and tigecycline had a significantly faster effect in eradicating biofilms than linezolid, rifampin, and vancomycin. Rifampin in combination with any of these antibiotics was significantly more effective in biofilm eradication than any antibiotic used alone; however, when used alone, rifampin led to rapid emergence of rifampin-resistant MRSA [79]. Antibiotic locks are not recommended as the preferred strategy given the high risk of resistance.

Implants and catheters coated with antiseptics (chlorhexidine and silver sulfadiazine) have been shown to inhibit *S. aureus*-associated biofilm formation. In a study, Sampath et al. used a murine model to compare the inhibitory properties of various catheters impregnated with minocycline on their luminal surfaces and rifampin on their exterior surfaces, catheters coated with silver sulfadiazine, chlorhexidine, and both on the external surface and in the lumens [80]. Both catheters inhibited the growth of *S. aureus* biofilms. However, the major concern of the antibiotic coated implants and catheters is the rapid emergence of resistant strains that can result from their use. Hence, the use of coated catheters is not encouraged.

### 4.3. Other Potential Approaches for Management of Biofilm Producing MRSA Infections—Road to the Future

Several bio-molecules are being investigated as adjunctive therapies and as novel anti-biofilm agents, including bacteriophages, metal chelators, phytochemicals, nanoparticles, repurposed drugs, antimicrobial peptides (AMPs), enzymes, and antibodies to inhibit or treat biofilms. These treatment modalities are briefly discussed as follows.

#### 4.3.1. Chelators and Sulfhydryl Compounds

Cations (e.g., Mg^2+^, Fe^2+^, Ca^2+^) play a crucial role in bacterial growth by promoting inter-bacterial interactions and aggregation and are thought to be important for microbial adherence and biofilm formation. By sequestering these ions, high-affinity metal ion chelators such as ethylenediamine tetra acetic acid (EDTA), ethylene glycol tetra acetic acid (EGTA), and tri-sodium citrate (TSC) inhibit biofilm formation as well as bacterial adhesion to surfaces, thus showing useful antibacterial properties in vitro [81]

#### 4.3.2. Nanoparticles

The application of nanotechnology to the field of biofilm inhibition is a novel area of scientific exploration. Nanoparticles (e.g., gold, silver, iron, copper and selenium), and nanomaterials have shown enhanced biofilm matrix penetration compared to free drug molecules [82], as such interfering with biofilm formation and bacterial adhesion. The field of nanotechnology provides novel approaches to tackle *S. aureus* biofilm-associated infections. Several nanomaterials, nanoparticles (NP), and drug encapsulated nanoparticles have been shown to possess better antibacterial and anti-biofilm activities. Because nanoparticles can interact with and penetrate the biofilm matrix more effectively than free drug molecules [61], they interfere with *S. aureus* adhesion and thus prevent its biofilm formation. Utilizing nanoparticles like silver and zinc oxide as an adjunctive therapy, given their enhanced biofilm penetrative properties to antibiotics, has been proposed [83].

A study showed Gold nanocage (AuNC@NO) to display potential application as a beneficial antibiofilm agent for treating biofilm-associated infections [84].

Gold nanocage (AuNC@NO) releases nitric oxide (NO), which is activated by near-infrared (NIR) irradiation to provide NO and produce hyperthermia for biofilm removal [84].

AuNC@NO has the qualities of delayed NO release at physiological temperature and on-demand fast NO release under NIR irradiation, as well as steady and good photothermal conversion efficiency [84].

Based on these characteristics, AuNC@NO displays in vitro bactericidal and antibiofilm performance and could eliminate 85.4% of biofilms and reduce bacteria by four orders of magnitude under NIR irradiation [84]. According to the in vivo results, NO release from AuNC@NO was significantly accelerated after 5 min of 0.5 W cm2 NIR irradiation, which led to the dispersal of MRSA biofilms and worked in conjunction with photothermal therapy (PTT) to kill planktonic MRSA that had lost its biofilm protection [84]. Due to the controlled photothermal temperature and toxicity, the surrounding tissues suffered little harm. This novel nanocomposite technology offers a promising therapeutic approach and needs further evaluation in clinical settings.

#### 4.3.3. Repurposed Drugs

Utilizing repurposed drugs, i.e., Food and Drug Administration (FDA)-approved drugs indicated for non-MRSA infections/autoimmune diseases, has been proposed for the treatment of biofilm infections. Some examples of these include niclosamide, a drug commonly used for treating Taenia (tapeworm) infections, thioridazine, which is an anti-psychotic agent, and auranofin, which is an antirheumatic agent, given their anti-biofilm activity shown in vitro [85]. However, their applicability in clinical settings needs to be further studied.

#### 4.3.4. Antimicrobial Peptides (AMPs)

These are peptides that are positively charged, amphipathic in nature; and composed of fewer than 50 amino acids in length. To date, more than 5000 AMPs have been described [86]. AMPs can bind to and disrupt bacterial membranes, and some of these AMPs possess anti-biofilm activity against *S. aureus*. Although larger than AMPs, human short-palate lung and nasal epithelial clone 1 (SPLUNC1) protein possess anti-biofilm activity against *S. aureus*. SPLUNC1 is a 256-amino acid multifunctional protein secreted by the human respiratory tract. SPLUNC1 helps in maintaining fluid homeostasis in airway epithelia and possesses antimicrobial activity [87]. Based on the sequences of naturally occurring antimicrobial peptides, synthetic peptides are thought to be a promising treatment option for bacterial infections that are resistant to standard antibiotics. Small, cationic peptides with a variety of antimicrobial and immunological activities are known as antimicrobial peptides or AMPs. One of the main human AMPs that is crucial to the body’s defense against both systemic and local infections is LL-37 [88]. A synthetic derivative of LL-37, designated OP-145 or P60.4Ac, which includes the core antimicrobial region of LL-37 has improved antimicrobial and similar endotoxin-neutralizing activities of LL-37. Recent studies have shown that OP-145, when incorporated in a biodegradable implant coating, can prevent *S. aureus*-induced biomaterial-associated infection in rabbits [89].

Another molecule SAAP-148 is able to prevent the formation of predominantly polysaccharide, as well as proteinaceous biofilms, and to promote their breakdown and eradicate established *S. aureus* biofilms. *S. aureus* persisters that survived an extremely high dose of rifampicin were completely eradicated within 2 h by SAAP-148 at low micromolar concentrations. This peptide rapidly interacts with and subsequently permeabilizes the cytoplasmic membrane of bacteria, leading to bacterial death. The powerful activity of SAAP-148 against dividing and nondividing, metabolically inactive bacteria living in a biofilm, as well as against persister cells, is consistent with the method of action comparable to that used by LL-37 and LL-37 derivatives. It has long been thought that because of this rapid, membrane-based mechanism of action, resistance development to AMPs is very unlikely [90]. Thus, AMPs might prove to be a promising adjunctive therapeutic option for treating biofilms, and research is actively ongoing in this area [91].

#### 4.3.5. Enzymes as Biofilm Disrupting Agents

Various enzymes have been identified to be effective against *S. aureus* biofilms. These can weaken *S. aureus* biofilms by destroying their cell wall or extracellular matrix, and include different cell wall hydrolases that can degrade the pentaglycine bridges in the *S. aureus* cell wall (e.g., lysostaphin, α-amylase, hyaluronidase, cysteine/histidine-dependent amidohydrolase/peptidase, endolysins) [92], proteases (e.g., V8 protease and cysteine proteases), and DNases.

Lysostaphin, a glycyl glycine endopeptidase that cleaves the pentaglycine cross-bridge of staphylococcal cell walls, has been shown to be able to lyse and disrupt the intricate structure of staphylococcal biofilms [93] and bacteriophage endolysins including cysteine/histidine-dependent amidohydrolase/peptidase from phage K (CHAPK), LysH5 from phage vB-SauS-phiIPLA88, and endolysin from phi11 phage have been found to possess the ability to lyse and disrupt the complex structure of staphylococcal biofilms [94].

In the quest for methods that can disrupt the biofilms, attempts have also been made to use proteases and nucleases encoded by staphylococci. Among the proteases, V8 protease was noted to inhibit *S. aureus* biofilm development and encourage biofilm detachment by inactivating autolysin (AtlA), and nucleases such as staphylococcal nuclease (Nuc), have also demonstrated anti-biofilm efficacy [95,96].

#### 4.3.6. Phytochemicals

Plant derived compounds, including different phytochemicals (e.g., tannic acid, ellagic acid, xanthohumol, etc.), several polyphenolic compounds, and flavonoids, exhibit a wide range of anti-inflammatory, anti-allergic, hepatoprotective, antithrombotic, anti-carcinogenic, and vasodilatory actions. Phytochemicals are thought to act by affecting bacterial quorum-sensing activity, thus interfering in the cell–cell interactions and in biofilm formation [97]. Phytochemicals including 7-hydroxycoumarin (7-HC), indole-3-carbinol (I3C), salicylic acid, and saponin were analyzed, and 7-HC and I3C were shown to be the most effective against biofilm-producing *S. aureus* [97]. Research has also looked at using antibiotics and phytochemicals together to combat *S. aureus* biofilms, and some examples that have showed synergism include combinations of oxacillin and xanthohumol; 13C and tetracycline, erythromycin and ciprofloxacin [97,98].

Most of the plant derivatives hold great promise to tackle *S. aureus* biofilm infections; however, they require further in vivo experimental validations as all the data obtained from plant-derived compounds are based on in vitro results.

#### 4.3.7. Staphylococcal Phages

Phages are naturally occurring viruses that infect bacteria. Bacteriophages are capable of killing antibiotic-resistant bacteria without harming commensals [99]. The mechanism of action of phage therapy involves penetration and degradation of extracellular biofilm by bacteriophages [100]. A phage can infect MRSA strains in both biofilm and planktonic phases, suggesting PAC regimens as effective adjuncts to antibiotics. It was shown that a triple combination of bacteriophage (Sb-1 phage), ceftaroline, and daptomycin effectively reduced *S. aureus* populations below detection limits, even in biofilm conditions, for several of the studied strains, irrespective of strain-specific MIC or growth stage [100]. Adjunctive intravenous phage therapy was studied in 13 patients with severe *S. aureus* infections and was noted to be well-tolerated [101] and the diSArm trial, which is a phase 1b/2a randomized trial studying the safety/efficacy of bacteriophage adjunctive therapy, is ongoing [58,102].

Phage cocktails have also been studied to target staphylococcal biofilms. For instance, Alves et al. demonstrated that a combination of *BacteriophageK* and *DRA88* (a broad host range phage) can effectively reduce *S. aureus* biofilm biomass within 48 h [103].

Thus, phage and antibiotic combinations (PAC) may prove to be more effective for treating biofilms than either type of agent alone and their potential use as concomitant therapies seems promising; however, widespread use warrants continued investigation in clinical scenarios.

#### 4.3.8. Surface Modifications of Medical Devices

As biofilm formation is influenced by the physical properties of the biomaterials, cell surface dynamics (including the hydrophobicity, topology, and electrostatic interactions) are an important determining factor in the attachment of staphylococci to biomaterial surfaces.

Increased surface smoothness enhanced *S. aureus* attachment, while increased surface roughness of the implant materials reduced *S. aureus* attachment [104], and *S. aureus* binding to implants was shown to be considerably decreased by nanopatterning titanium oxide to create rough implant surfaces [105].

#### 4.3.9. Laser Shock Waves (LSW), Ultra Sound (US), and Photodynamic Therapy (PDT)

Ultra sound is an oscillating sound that is above the range of human hearing, and laser shock waves are high energy waves moving at supersonic speed. Both of these methods were shown to be effective at breaking up biofilms, enhancing antibiotic therapy [106]. Using a specific wavelength of light, photodynamic therapy activates photosensitizing agents and produces reactive oxygen species that are harmful to bacteria and Staphylococcal biofilms. These photosensitizers include malachite green, methylene blue, sinoporphyrin sodium, toluidine blue O, chlorin e6, and 5-aminolevulinic acid [107,108,109].

#### 4.3.10. Antibodies/Vaccine Candidates

Vaccine/antibody development for *S. aureus* has been challenging, given the complex nature of staphylococcal infections and the production of a multitude of virulence factors [110]. Although attempts are being made to use capsular polysaccharide (type 5 and 8), clumping factors A and B, fibronectin binding protein, adenosine triphosphate binding cassette transporter, and amidase as potential vaccine candidates, and clumping factor A, adenosine triphosphate binding cassette transporter, and teichoic acids as therapeutic antibodies [111] to prevent and treat *S. aureus* infections, these are largely experimental. Several vaccine candidates have been proposed and most of the ones being investigated are antigen-based [110]. One of the vaccine candidates (rFSAV), composed of five recombinant *S. aureus* antigens (Hla, SEB, MntC, IsdB, and SpA), has shown promising efficacy in preclinical murine models [112,113]. Another heptavalent vaccine consisting of seven *S. aureus* toxoids, named IBT-V02, has also been shown to be effective in animal models [113,114,115]. Epitope-based vaccine strategy is also being investigated for vaccine production, and immunization with two *S. aureus* vaccine candidates, coproporphyrinogen III oxidase (CgoX) and triose phosphate isomerase (TPI), which are essential for heme synthesis and glycolysis, respectively, has been shown to elicit protective immunity against *S. aureus*. Monoclonal antibodies against these antigens were also shown to be protective against *S. aureus* infection in mice [116]. Research in this area has been largely pre-clinical [117], and investigations are ongoing to develop a vaccine that would be effective in clinical scenarios.

Although endeavors are underway, more efforts are needed in this direction to design clinically successful vaccines or therapeutic antibodies against *S. aureus* biofilm infections.

## 5. Conclusions

MRSA continues to be a formidable pathogen, as a cause of prolonged and difficult to treat infections, with various protective mechanisms including biofilm production facilitating disease pathogenesis. MRSA infections cause high morbidity and mortality in all age groups and are a serious therapeutic challenge. While present clinical guidelines emphasize prompt debridement, removal of primary infection foci/infected hardware, and the use of antibiotics, novel approaches to fight this ubiquitous pathogen are needed. Dual antimicrobial therapy is often needed for persistent bacteremia and metastatic infections, and antimicrobial therapy combinations that include rifampin with its property of biofilm penetration have been demonstrated to be useful, especially while treating serious MRSA infections such as endocarditis, central nervous system infections, or osteomyelitis. Studies have investigated the potential roles of different bacteriophages, phytochemicals, nanoparticles, antibodies, metal chelators, enzymes, and even ultra sound or shock waves/photodynamic therapy for eradication of biofilms. Although many promising newer agents and combination approaches for treatment of biofilms have been described and are being studied as adjunctive therapeutics, their role and practical application in real world settings needs to be clarified. Further studies evaluating clinical use and applicability of these novel treatment strategies are needed. Until then, antibiotic therapy with infected device /hardware removal and debridement of infected tissue remain the mainstays of treatment.

## Figures and Tables

**Table 1:** Antibiotics for MRSA infections: Class, mechanism of action, route, adverse drug reaction profile, and pharmacokinetics [46,54,55,56].

Medication	Class	Mechanism of Action	Route	Adverse Drug Reactions	Pharmacokinetics
Vancomycin	Glycopeptide	Inhibits bacterial wall synthesis by binding to D-alanyl-D-alanine and blocking glycopeptide polymerization.	IV, PO, Rectal	Common: Hypokalemia, Abdominal pain, Diarrhea, Nausea/vomiting Serious: Hypotension, Clostridioides difficile diarrhea, Agranulocytosis, Neutropenia, Thrombocytopenia, Anaphylaxis, Ototoxicity, Nephrotoxicity	Absorption: IV 100%. Oral/rectal: negligible Distribution: Widely distributed. Crosses blood–brain barrier Vd: 0.2 L/kg to 1.25 L/kg Metabolism: None Excretion: IV = renal, PO = fecal unchanged, dialyzable Elimination half-life: 4 to 6 h in healthy adults, pediatrics 5 to 21 h depending on age
Rifampin	Rifamycin	Binds to the beta subunit of DNA-dependant RNA polymerase, blocking RNA transcription and bacterial RNA synthesis.	Oral, IV	Common: None Serious: Hepatotoxicity, Anaphylaxis, Nephrotoxicity, Interstitial lung disease, Mycobacteriosis, Agranulocytosis, Disseminated intravascular coagulation, Thrombotic microangiopathy	Absorption: IV 100%. Well-absorbed orally, food may delay absorption. Distribution: Lipophilic. Crosses blood–brain barrier Vd: 0.66 L/kg Metabolism: Hepatic Excretion: Feces 60–65%, urine around 30%, non-dialyzable. Elimination half-life: Infants and children = 1–4 h, Adults = 2–3 h
Daptomycin	Cyclic Lipopeptide	Inhibits intracellular synthesis of DNA, RNA, and protein. Causes rapid cell wall depolarization of susceptible organisms.	IV	Common: Fever, Dyspnea, Pain in throat, Dizziness, Headache, Insomnia, Abdominal pain, Diarrhea, Vomiting, Pruritis, Rash, Hypertension, Hypotension Serious: Increase in creatinine kinase level, Rhabdomyolysis, Renal failure, Pulmonary eosinophilia	Absorption: IV 100%. Distribution: Widely distributed. Inactivated by lung surfactants. Crosses blood–brain barrier Vd: 0.1 L/kg Metabolism: Negligible Excretion: 78% renally unchanged, 5.7% fecally unchanged, dialyzable Elimination half-life: 8 h in adults, 4.4–7.5 h in pediatric patients
Ceftaroline	Cephalosporin 5th Generation	Binds to penicillin-binding proteins which inhibit bacterial cell wall synthesis.	IV	Common: Rash, Fever, Diarrhea, Nausea/vomiting Serious: Clostridioides difficile diarrhea, Elevation in ALT/SGPT level, Anaphylaxis, Encephalopathy, Seizure	Absorption: IV 100% Distribution: Widely distributed Vd: 20.5 L Metabolism: To active drug in plasma by a phosphatase enzyme Excretion: 88% renal, 6% fecal, dialyzable Elimination half-life: 1–3 h
Clindamycin	Lincosamide	Inhibits bacterial protein synthesis by binding to 50S ribosomal subunits.	IV, PO, Topical, Vaginal	Common: Xeroderma, Nausea, Diarrhea, Morbilliform eruption. Serious: Erythema multiforme, Stevens–Johnson syndrome, Toxic epidermal necrolysis, Acute kidney injury, Anaphylaxis, Agranulocytosis, Clostridioides difficile infection, Hemorrhagic diarrhea	Absorption: IV 100%, Oral 90%, vaginal cream 5%, vaginal suppository 30% Distribution: Widely distributed, does not cross blood–brain barrier Vd: 0.6–1.2 L/kg Metabolism: Metabolized to active form primarily by CYP3A4 Excretion: 10% renally unchanged, 3% fecally unchanged, non-dialyzable Elimination half-life: 4–6 h in neonates, 2 h in infants and pediatric patients, 3 h in adults
Linezolid	Oxazolidinone	Binds to bacterial 23S ribosomal RNA of the 50S subunit to inhibit bacterial protein synthesis.	IV, PO	Common: Headache, Nausea/vomiting, Diarrhea Serious: Serotonin syndrome, Disorder of optic nerve, Peripheral neuropathy, Seizure, Hepatic injury, Myelosuppression, Clostridioides difficile infection, Hyponatremia, Lactic acidosis, Syndrome of inappropriate antidiuretic hormone secretion	Absorption: IV 100%, oral 100% Distribution: Widely distributed. Crosses blood–brain barrier. Vd: 0.65 L/kg Metabolism: Hepatic Excretion: Urine (30% unchanged, 50% as metabolites), 9% feces as metabolites, dialyzable. Elimination half-life: 5 h
Tedizolid	Oxazolidinone	Binds to bacterial 50S RNA subunit to inhibit bacterial protein synthesis.	IV, PO	Common: Headache, Dizziness, Diarrhea, Nausea/vomiting Serious: Disorder of optic nerve, Neutropenia, Peripheral nerve disease, Clostridioides difficile infection, Colitis, Tachycardia	Absorption: IV 100%, oral 91%. Distribution: Plasma, adipose, and skeletal muscle tissue Vd: 67–80 L Metabolism: Converted to active metabolite by phosphatases Excretion: 82% feces, 18% urine, nondialyzable Elimination half-life: 12–17 years old = 7 h, Adults: 12 h
Bactrim	Sulfonamide Derivative	Interferes with bacterial folic acid pathways.	IV, PO	Common: Rash, Urticaria, Nausea/vomiting, Loss of appetite Serious: Prolonged QT interval, Torsades de pointes, Ventricular tachycardia, Acute kidney injury, Rhabdomyolysis, Anaphylaxis, Hepatic necrosis, Agranulocytosis, Aplastic anemia, Neutropenia, Thrombocytopenia, Clostridioides difficile infection, Hyponatremia, Stevens–Johnson syndrome, Sweet’s syndrome, Toxic epidermal necrolysis, Erythema multiforme	Absorption: IV 100%, oral 90–100% Distribution: Widely distributed. Crosses blood–brain barrier Vd: 2.7 L/kg in neonates, 1.5 -0.86 L/kg in infants and children, 1.3 L/kg in adults Metabolism: Hepatic Excretion: Urine as metabolites and unchanged drug, dialyzable Elimination half-life: 19 h in neonates, 4 h in infants and children 1–10 yo, 8 h in children > 10 yo and adults
Oritavancin	Glycopeptide	Binds to stem peptides of peptidoglycan precursors which inhibits cell wall biosynthesis.	IV	Common: Nausea/vomiting, Abscess, Headache Serious: Clostridioides difficile infection, Infusion reaction, Cellulitis, Osteomyelitis, Hypersensitivity reaction, Hemorrhage, Elevated INR, Prothrombin time increased, Abnormal aPTT	Absorption: IV 100% Distribution: Extensive in skin, Vd indicates extensive distribution into tissues Vd: 87.6 L Metabolism: None Excretion: 5% urine and 1% feces as unchanged drug, non-dialyzable Elimination half-life: 245 h
Telavancin	Glycopeptide	Inhibits bacterial wall synthesis by binding to D-alanyl-D-alanine and blocking glycopeptide polymerization. Additionally, disrupts membrane potential and cell permeability.	IV	Common: Abnormal urine, Altered sense of taste, Nausea/vomiting Serious: Acute renal failure, Anaphylaxis, Prolonged QT interval	Absorption: IV 100% Distribution: Extensively through skin and pulmonary tissues. Vd: 0.13 L/kg Metabolism: Minimal, unknown mechanism. Excretion: 76% urine, dialysis affects on drug not studied (not recommended). Elimination half-life: 7 h
Dalbavancin	Glycopeptide	Inhibits bacterial wall synthesis by binding to D-alanyl-D-alanine and blocking glycopeptide polymerization.	IV	Common: Diarrhea, Nausea, Fever, Headache Serious: Hypersensitivity reaction, Elevated ALT/SGPT, Clostridioides difficile infection, Gastrointestinal hemorrhage	Absorption: IV 100% Distribution: Extensive in skin Vd: 9L Metabolism: Minimal Excretion: Urine 33% as unchanged drug and 12% as metabolite, 20% feces, nondialyzable. Elimination half-life: 346 h
Doxycycline	Tetracycline	Inhibits protein synthesis by binding with ribosomal subunits.	IV, PO	Common: Bacterial vaginosis, Myalgia, Diarrhea, Nausea/vomiting, Sensitive dentin, Rash Serious: Pseudotumor cerebri, Arrest of bone development/growth, Hepatotoxicity, Anaphylaxis, Clostridioides difficile infection, Stevens–Johnson syndrome, Toxic epidermal necrolysis.	Absorption: IV 100%, Oral 100%, reduced at high pH Distribution: Extensive into body tissues and fluids. CSF penetration is poor Vd: 1.36 L/kg Metabolism: Chelated in GI tract Excretion: 40% renal, 30% feces, non-dialyzable Elimination half-life: 19 h
Minocycline	Tetracycline	Inhibits protein synthesis by binding with ribosomal subunits.	IV, PO	Common: Fatigue, Dizziness, Headache Serious: Acidosis, Hyperphosphatemia, Clostridioides difficile infection, Enamel hypoplasia, Tooth discoloration, Autoimmune hepatitis, Hepatic failure, Anaphylaxis, Systemic lupus erythematosis, Arrest of bone developement/growth, Lightheadedness, Pseudotumor cerebri, Azotemia, Elevation of BUN, Serum sickness	Absorption: IV 100%, oral well absorbed Distribution: Extensive into body tissues and fluids. CSF penetration is poor Vd: 0.14 to 0.7 L/kg Metabolism: Hepatic Excretion: 10% urine, 28% feces, non-dialyzable Elimination half-life: 16 h
Tigecycline	Glycycline	Inhibits protein synthesis by binding with ribosomal subunits.	IV	Common: Headache, Abdominal Pain, Diarrhea, Nausea/vomiting Serious: Pseudotumor cerebri, All-cause death, Anaphylaxis, ALT/SGPT elevation, Hepatic disorder, Liver failure, Clostridioides difficile infection, Pancreatitis, Septic shock	Absorption: IV 100% Distribution: Extensive in tissues, no data on CNS penetration Vd: Children 8–11 yo = 2.84 L/kg, 8 L/kg in adults Metabolism: Hepatic Excretion: 59% feces, 33% urine, poorly dialyzed Elimination half-life: 27 h after single dose, 42 h after multiple doses
Eravacycline	Tetracycline	Inhibits protein synthesis by binding with ribosomal subunits.	IV	Common: Nausea/vomiting, Infusion reaction Serious: Azotemia, BUN elevation, Pseudotumor cerebri, Photosensitivity, Acidosis, Hyperphosphatemia, Clostridioides difficile infection, Necrosis of pancreas, Pancreatitis, Staining of teeth, Anaphylaxis, Arrest of bone development/growth	Absorption: IV 100% Distribution: Extensive into body tissues, no data on CNS penetration Vd: 4 L/kg Metabolism: CYP3A4 Excretion: 34% urine, 47% feces, literature does not state information about dialysis Elimination half-life: 20 h
Omadacycline	Tetracycline	Inhibits protein synthesis by binding with ribosomal subunits.	IV, PO	Common: Headache, Insomnia, ALT/SGPT elevation, AST elevation, Constipation, Diarrhea, Nausea/vomiting, Infusion reaction Serious: Photosensitivity, Acidosis, Hyperphosphatemia, Clostridioides difficile infection, Pancreatitis, Staining of teeth, Azotemia, BUN elevation, Arrest of bone development/growth, Anaphylaxis, Abnormal liver function	Absorption: IV 100%, oral 34.5%, food decreases absorption Distribution: Extensive in lung and skin, no data on CNS penetration Vd: 190 L Metabolism: Not metabolized Excretion: IV 27% urine, oral 80% feces Elimination half-life: 16 h
Fosfomycin	Miscellaneous	Inhibits pyruvyl transferase which in turn inhibits bacterial wall synthesis.	PO	Common: Pain, Pharyngitis, Rhinitis, Dysmenorrhea, Headache, Backache, Diarrhea, Nausea Serious: Aplastic anemia, Angioedema, Cholestatic jaundice, Hepatic necrosis, Toxic megacolon	Absorption: 37% oral Distribution: Primarily bladder tissue Vd: 1.5–2.4 L/kg Metabolism: Unknown Excretion: 38% oral, 18% feces, dialyzable Elimination half-life: 5 h
Quinupristin/Dalfopristin	Streptogramin	Binds to 50S bacterial ribosomal subunit which in turn inhibits bacterial protein synthesis.	IV	Common: Injection site disorders, Diarrhea, Nausea/vomiting, Thrombophlebitis, Arthralgia, Myalgia, Conjugated hyperbilirubinemia, Hyperbilirubinemia Serious: Generalized Myasthenia	Absorption: 100% IV Distribution: Extensive, including CNS penetration Vd: Quinupristine: 0.45 L/kg, dalfopristin 0.24 L/kg Metabolism: Hepatic, blood Excretion: 75% feces, 16% urine, suspected non-dialyzable based on molecule size Elimination half-life: quinupristin = 0.85 h, dalfopristin = 0.7 h
Delafloxacin	Fluoroquinolone	Inhibits DNA replication, repair, recombination, and transcription by inhibiting DNA topoisomerases.	IV, PO	Common: Diarrhea, Nausea/vomiting, ALT/SGPT elevation, AST elevation, Headache Serious: Agitation, Delirium, Disorientation, Memory impairment, Peripheral neuropathy, Pseudotumor cerebri, Raised intracranial pressure, Seizure, Myasthenia gravis, Rupture of tendon, Tendinitis, Hypersensitivity reaction, Hypoglycemia, Aortic aneurysm or dissection	Absorption: 100% IV, 58.8% oral Distribution: Extensive in skin and lung Vd: 30–48 L Metabolism: Hepatic Excretion: IV = 65% urine, 28% feces; oral = 50% urine, 48% feces, dialyzable Elimination half-life: IV = 3.7 h, oral = 6 h
Lefamulin	Pleuromutilin	Interacts with peptidyl transferases on ribosomal RNA of the 50S subunit.	IV, PO	Common: Headache, Insomnia, Elevated liver enzymes, Diarrhea, Nausea/vomiting, Hypokalemia, Injection site disorder Serious: Prolonged QT interval, Clostridioides difficile infection	Absorption: 100% IV, 25% oral (decreases slightly with food) Distribution: Extensive in lung Vd: 86.1 L Metabolism: Hepatic via CYP3A4 Excretion: IV = 77% feces, 15.5% urine. Oral = 89% feces, 5.3% urine Elimination half-life: 8 h, prolonged in patients with hepatic impairment

**Table 2:** Antibiotics for MRSA infections: Dosing by MRSA indication [46,54,55,56].

Medication	Adult Dose (MRSA Indications Only)	Pediatric Dose (MRSA Indications Only)	Neonatal Dose (MRSA Indications Only)
Vancomycin	Bacteremia, Cerebrospinal Fluid Shunt Infection (off-label use), Diabetic Foot Infection (off-label use), Endocarditis, Intracranial Abscess (off-label use), Meningitis (off-label use), Osteomyelitis, Pneumonia, Prosthetic Joint Infection, Sepsis/Septic Shock, Septic Arthritis, SSTI, Staph Toxic Shock Syndrome: 10–20 mg/kg IV every 8 to 48 h in adults. May consider a bolus of 20–35 mg/kg for seriously ill patients. Requires pharmacokinetic calculator and AUC monitoring to guide proper dose. Generally AUC values of around 400 are considered appropriate for most indications. Higher AUC (closer to 600) may be targeted for patients with meningitis or endocarditis. Requires renal dose adjustment.	Meningitis: 1 mo–18 yo: 15 mg/kg/dose IV every 6 h. Serious MRSA Infection Treatment: 3 mo–11 yo: 60–80 mg/kg/day IV divided every 6 h (max dose: 3600 mg/day). 12 yo–18 yo: 60–70 mg/kg/day IV divided every 6–8 h (max dose: 3600 mg/day). Recommend AUC monitoring to guide proper dose. Generally AUC values of around 400 are considered appropriate for most indications. Higher AUC (closer to 600) may be targeted for patients with meningitis or endocarditis. If trough values are utilized, values of around 5–15 mg/L are recommended, staying as close to 10 mg/L as possible. Requires renal dose adjustment.	Serious MRSA Infections: 10–20 mg/kg IV. Frequency dependent on post-menstrual age (PMA) and postnatal age (PNA). Recommend AUC monitoring to guide proper dose. Generally AUC values of around 400 are considered appropriate for most indications. Higher AUC (closer to 600) may be targeted for patients with meningitis or endocarditis. If trough values are utilized, values of around 5–15 mg/L are recommended, staying as close to 10 mg/L as possible. Requires renal dose adjustment.
Rifampin	Staphylococcal Synergy (off-label): 300–600 mg IV or orally every 12 h in combination with other agents. May require renal dose adjustment.	Endocarditis Synergy: 1 mo–18 yo: 15 mg/kg/day IV or orally divided every 8 h (max dose: 900 mg/day). May require renal dose adjustment.	Staphylococcal Infections: Oral: 10–20 mg/kg/dose every 24 h. IV: 5–10 mg/kg/dose every 12 h. May require renal dose adjustment.
Daptomycin	Bacteremia: 6–12 mg/kg IV every 24 h. Cerebrospinal fluid shunt infection (off-label use), Intracranial Abscess (off-label use), Meningitis (off-label use), Osteomyelitis and/or Discitis (off-label use), Prosthetic Joint Infection (off-label use), Septic arthritis (off-label use): 6–10 mg/kg IV Q 24 h. Diabetic Foot Infection (off-label use), SSTI: 4–6 mg/kg IV every 24 h. Endocarditis: 8–12 mg/kg IV every 24 h. Requires renal dose adjustments. Do not use for the treatment of pneumonia.	Bacteremia: 1 mo-6 yo: 12 mg/kg/dose IV every 24 h. 7 yo–11 yo: 9 mg/kg/dose IV every 24 h. 12 yo–17 yo: 7 mg/kg/dose IV every 24 h. Endocarditis: 1 mo–5 yo: 10 mg/kg/dose IV every 24 h. 6 yo–18 yo: 6 mg/kg/dose IV every 24 h. Osteomyelitis: 1 mo–5 yo: 12 mg/kg/dose IV every 24 h. 7 yo–11 yo: 9 mg/kg/dose IV every 24 h. 12 yo–17 yo: 7 mg/kg/dose IV every 24 h. SSTI: 1 yo–23 mo: 10 mg/kg/dose IV every 24 h. 2 yo–5 yo: 9 mg/kg/dose IV every 24 h. 7 yo–11 yo: 7 mg/kg/dose IV every 24 h. 12 yo–17 yo: 5 mg/kg/dose IV every 24 h. Requires renal dose adjustment. Do not use for the treatment of pneumonia.	Dosing guidelines not available.
Ceftaroline	Bacteremia (off-label use): 600 mg IV every 8 h. Community Acquired Pneumonia, Hospital acquired pneumonia (off-label use), SSTI: 600 mg IV every 12 h. Requires renal dose adjustment.	Pneumonia: 2 mo–23 mo: 8 mg/kg/dose IV every 8 h. 2 yo–17 yo (≤33 kg): 12 mg/kg/dose IV every 8 h. 2 yo–17 yo (>33 kg): 400 mg IV every 8 h or 600 mg IV every 12 h. SSTI: <2 mo: 6 mg/kg/dose IV every 8 h. 2 mo–23 mo: 8 mg/kg/dose IV every 8 h. 2 yo–17 yo (≤33 kg): 12 mg/kg/dose IV every 8 h. 2 yo–17 yo (>33 kg): 400 mg IV every 8 h or 600 mg IV every 12 h. Requires renal dose adjustment.	SSTI: PMA 34 weeks or greater: 6 mg/kg/dose IV every 8 h. Requires renal dose adjustment.
Clindamycin	Diabetic Foot Infection (off-label use): 300–450 mg orally every 6–8 h. MRSA Osteomyelitis: 600–900 IV every 8 h, 600 mg orally every 8 h. Pneumonia, Septic arthritis: 600 mg IV or orally every 8 h. Prosthetic Joint Infection (off-label use): 600 mg orally every 8 h. SSTI: 300 mg orally 4 times daily, 600–900 mg IV every 8 h. Toxic Shock Syndrome, Toxin Production Suppression: 900 mg IV every 8 h in combination with other agents.	Pneumonia: 3 mo–18 yo: 40 mg/kg/day IV divided every 6–8 h (max dose: 2700 mg/day). SSTI (Impetigo): 3 mo–18 yo: 20 mg/kg/day orally divided every 8 h (max dose: 400 mg/dose). SSTI: 3 mo–18 yo: 30–40 mg/kg/day orally in divided doses every 6–8 h (max dose: 450 mg/dose). Toxic Shock Syndrome, Toxin Production Suppression: 3 mo–18 yo: 40 mg/kg/day IV divided every 6–8 h (max dose: 900 mg/dose).	Postmenstrual Age 32 weeks or less: 5 mg/kg IV. Postmenstrual Age 32–40 weeks: 7 mg/kg IV. Frequency dependent on post-menstrual age and postnatal age.
Linezolid	Bacteremia (off-label use), CNS infection (off-label use), Diabetic Foot Infection, Endocarditis (off-label use), Intracranial/Spinal Epidural, Brain Abscess, Meningitis (off-label use), Osteomyelitis (off-label use), Pneumonia, Prosthetic Joint Infection (off-label use), Septic Arthritis (off-label use), SSTI, Toxic Shock Syndrome (off-label use): 600 mg IV or orally every 12 h.	Bacteremia, Endocarditis, Meningitis, Pneumonia, SSTI (complicated): 1 mo–11 yo: 10 mg/kg/dose IV or orally every 8 h (max dose: 600 mg/dose). 12 yo–18 yo: 600 mg IV or orally every 12 h. SSTI (Uncomplicated): 1 mo–5 yo: 10 mg/kg/dose IV or orally every 8 h (max dose: 600 mg/dose). 5 yo–11 yo: 10 mg/kg/dose IV or orally every 12 h (max dose: 600 mg/dose). 12 yo–18 yo: 600 mg IV or orally every 12 h.	Pneumonia by Post Natal Age (PNA) GA < 34 weeks: PNA < 7 days: 10 mg/kg/dose IV or orally every 8–12 h. PNA ≥ 7 days: 10 mg/kg/dose IV or orally every 8 h. GA ≥ 34 weeks: 10 mg/kg/dose IV or orally every 8 h. SSTI: GA < 34 weeks: PNA < 7 days: 10 mg/kg/dose IV or orally every 8–12 h. PNA ≥ 7 days: 10 mg/kg/dose IV or orally every 8 h. GA ≥ 34 weeks: 10 mg/kg/dose IV or orally every 8 h.
Tedizolid	SSTI: 200 mg IV or orally every 24 h.	SSTI: 12 yo–18 yo: 200 mg IV or orally Q 24 h.	Dosing guidelines not available.
Bactrim	Diabetic Foot Infection (off-label use): 2 double strength tablets orally every 12 h. Brain Abscess, Inracranial/Epidural Abscess (off-label use): 5mg/kg of trimethoprim component IV every 8–12 h. Meningitis (off-label use): 5 mg/kg of trimethoprim component IV every 6–12 h. Osteomyelitis (off-label use): 4 mg/kg of trimethoprim component IV or orally every 12 h with rifampin. Prosthetic Joint Infection (off-label use): 1 double strength tablet orally every 12 h with rifampin. Septic Arthritis (off label use): 2 double strength tablets orally every 12 h, 4 mg/kg trimethoprim component IV every 12 h. SSTI, Impetigo (off-label use): 1–2 double strength tablets orally every 12 h. Requires renal dose adjustment.	Meningitis: 2 mo–18 yo: 10–20 mg/kg/day of trimethoprim component IV every 6–12 h. SSTI: 2 mo–18 yo: 8–12 mg/kg/day of trimethoprim component IV/orally every 6–12 h (max dose: 320 mg/dose). Requires renal dose adjustment.	Dosing guidelines not available.
Oritavancin	SSTI: 1.2 g IV once.	Dosing guidelines not available.	Dosing guidelines not available.
Telavancin	Bacteremia (off-label use), Hospital Acquired Pneumonia, SSTI: 10 mg/kg IV Q 24 h. Requires renal dose adjustment.	Dosing guidelines not available.	Dosing guidelines not available.
Dalbavancin	SSTI: 1.5 g IV once, then 500 mg IV once 7 days later. Requires renal dose adjustment.	SSTI: 1 mo–6 yo: 22.5 mg/kg IV once (max dose = 1500 mg/dose). 6 yo–18 yo: 18 mg/kg IV once (max dose = 1500 mg/dose). Requires renal dose adjustment.	Acute Bacterial Skin and Skin Structure Infection: 22.5 mg/kg IV once.
Doxycycline	SSTI: 100 mg orally twice daily in combination with another agent.	Community Acquired MRSA SSTI: 8 yo–18 yo: ≤45 kg: 2 mg/kg/dose orally every 12 h. >45 kg: 100 mg orally twice daily.	Dosing guidelines not available.
Minocycline	Prosthetic Joint Infection (continuation after initial IV therapy for MRSA) (off-label use): 100 mg orally every 12 h. SSTI: 100 mg orally every 12 h.	Community Acquired MRSA SSTI: 9 yo–18 yo: 4 mg/kg (max dose: 200 mg) orally once, then 2 mg/kg/dose (max dose: 100 mg) orally every 12 h.	Dosing guidelines not available.
Tigecycline	SSTI: 100 mg IV once, then 50 mg IV every 12 h. Requires dose adjustment for hepatic impairment.	General Dosing Guidelines for Susceptible Infections: 1 mo–7 yo: 1.5–3 mg/kg IV once, then 1–2 mg/kg/dose IV every 12 h (max dose 50 mg/dose). 8 yo–11 yo: 1.2–2 mg/kg IV every 12 h (max dose = 50 mg/dose). 12 yo–18 yo: 50 mg IV every 12 h. Last line therapy for <8 yo due to impact on tooth development. Requires dose adjustment for hepatic impairment.	Dosing guidelines not available.
Eravacycline	Intra-abdominal Infection: 1 mg/kg IV every 12 h. Requires dose adjustment for hepatic impairment.	Dosing guidelines not available.	Dosing guidelines not available.
Omadacycline	SSTI: 200 mg IV once on day 1, then 100 mg IV every 24 h. 450 mg orally once on day 1, then 300 mg orally every 24 h.	Dosing guidelines not available.	Dosing guidelines not available.
Fosfomycin	Cystitis: 3 g orally once. Requires renal dose adjustment.	UTI: <12 years: 2000 mg orally once. 12–18 yo: 3000 mg orally once. Requires renal dose adjustment.	Dosing guidelines not available.
Quinupristin/Dalfopristin	Bacteremia (off-label use), CNS infection (off-label use): 7.5 mg/kg IV every 8 h. SSTI: 7.5 mg/kg IV every 12 h.	(1 mo-18 yo) MRSA Salvage Therapy: 7 mg/kg/dose every 8 h.	MRSA Salvage Therapy: 7.5 mg/kg/dose IV every 12 h.
Delafloxacin	Pneumonia (off-label use), SSTI: 300 mg IV every 12 h. 450 mg orally every 12 h. Requires renal dose adjustment.	Dosing guidelines not available.	Dosing guidelines not available.
Lefamulin	Pneumonia: 600 mg orally every 12 h, 150 mg IV every 12 h. Requires hepatic dose adjustment.	Dosing guidelines not available.	Dosing guidelines not available.

## Data Availability

Data are contained within the article.
